# At the intersection of lay and professional social networks: how community ties shape perceptions of mental health treatment providers

**DOI:** 10.1017/gmh.2015.25

**Published:** 2016-02-04

**Authors:** B. L. Perry, E. Pullen, B. A. Pescosolido

**Affiliations:** 1 Department of Sociology, Indiana University, Bloomington, IN 47405, USA; 2 Indiana University Network Science Institute, Bloomington, IN 47405, USA

**Keywords:** Community ties, mental health, networks, therapeutic alliance, physician-patient relationship, mental health treatment

## Abstract

**Background.:**

The therapeutic alliance is a critical determinant of individuals’ persistence and outcomes in mental health treatment. Simultaneously, individuals’ community networks shape decisions about whether, when, and what kind of treatment are used. Despite the similar focus on social relationship influence for individuals with serious mental illness, each line of research has maintained an almost exclusive focus on either ‘inside’ (i.e. treatment) networks or ‘outside’ (i.e. community) networks, respectively.

**Method.:**

For this study, we integrate these important insights by employing a network-embedded approach to understand the therapeutic alliance. Using data from the Indianapolis Network Mental Health Study (INMHS, *n* = 169, obs = 2206), we target patients experiencing their first major contact with the mental health treatment system. We compare patients’ perceptions of support resources available through treatment providers and lay people, and ask whether evaluations of interpersonal dimensions of the therapeutic alliance are contingent on characteristics of community networks.

**Results.:**

Analyses reveal that providers make up only 9% of the whole social network, but are generally perceived positively. However, when community networks are characterized by close relationships and frequent contact, patients are significantly more likely to report that treatment providers offer useful advice and information. Conversely, when community networks are in conflict, perceptions of treatment providers are more negative.

**Conclusion.:**

Community-based social networks are critical for understanding facilitators of and barriers to effective networks inside treatment, including the therapeutic alliance. Implications for community-based systems of care are discussed in the context of the USA and global patterns of deinstitutionalization and community reintegration.

There is agreement in the mental health services literature that a holistic community support system – which provides mental health treatment in conjunction with support for peer and family relationships and resources – is critical for recovery (Anthony & Blanch, 1989; Stroul, 1989). Likewise, research suggests that mental health services that involve lay members of social networks, such as behavioral family interventions, contribute to reducing relapse and readmission and improving medication compliance among individuals with serious mental illness (Tarrier *et al*. 1989; Pitschel-Walz *et al*. 2001). While existing models of community-based care emphasize the importance of developing a social context that is conducive to recovery, the focus has primarily been on engaging family members and augmenting informal support resources. Moreover, the therapeutic alliance and work accomplished in therapy is typically viewed as influencing social functioning within family and peer relationships rather than the reverse process.

Two essential groups of ‘caregivers’ play a critical role in recovery from an episode of mental illness, especially as the treatment system becomes increasingly community based. First, mental health providers build a therapeutic alliance with clients, which facilitates the establishment of common recovery goals and affects positive change in people's lives (Cruz & Pincus, 2002; Adams *et al*. 2007). Second, lay members of community-based networks provide advice, information, and opinions that influence whether or not individuals with mental health problems contact the mental health system and how they experience treatment (Pescosolido *et al*. 1998*a*; Carpentier & Bernard, 2011; Abbott *et al*. 2012; Schafer, 2013; Perry & Pescosolido, 2015). In essence, the former are ‘inside networks’ built in treatment while the latter describes ‘outside networks’ in the community (Pescosolido, 2006).

Yet, while both approaches focus on the critical role of social ties in the response to mental illness, any empirical connection between them is largely absent. Moreover, individuals may not divide their experiences and the important people in their lives along these artificial lines. While research on the therapeutic alliance looks to the influence of the individual and dyadic characteristics of treatment providers and clients, research on pathways to care looks to the influence of broader social and cultural contexts in which clients are embedded. However, if the availability of emotional and informational support through informal networks influences a clients’ perception of treatment, the spillover to adherence and continuity of care may represent an unacknowledged element of the recovery process.

To examine this possibility, we use a network-embedded approach, examining how social networks in the community – their outside networks – are associated with clients’ perceptions of supportive aspects of their relationship with mental health treatment providers – their inside networks. Using data from the Indianapolis Network Mental Health Study (INMHS, *n* = 169), we focus on a sample of patients making their first major contact with the mental health treatment system. Couched in the network framework, we identify characteristics of egos (i.e. persons with mental health problems), alters (i.e. those individuals that persons with mental health problems interact with regularly), ties (i.e. characteristics of relationships clients name), and network context (i.e. the aggregate characteristics and functions of each individual's social network). We examine whether and how perceptions of relationships with mental health treatment providers are contingent not only on the provider's and client's own characteristics, but also on the broader personal community-based social networks of the client.

## Therapeutic alliance: evolution and impact

While the terminology has changed over time, the importance of the relationship between those in need of mental health treatment and those that provide care has remained steadfast. Interpersonal processes that occur between physicians and patients, providers and clients, or practitioners and consumers are termed the therapeutic alliance and has long been of substantive interest in health research (Truog, 2012). While studies document the importance of patient perceptions of physicians in primary care practice (Browne *et al*. 2010), pediatric care (Tanner *et al*. 2009), and oncology (Fallowfield, 2008), in no other field is the therapeutic alliance considered to be more vital to positive outcomes than in treating serious mental illness (Laugharne & Priebe, 2006). While conceptualization and measurement of the therapeutic alliance has varied widely (Elvins & Green, 2008), most agree that it contains two dimensions: Task related alliance and personal alliance (Green, 2006). The former refers to contractual aspects of treatment planning, goal orientation, task understanding, and investment in therapy. The latter typically addresses the interpersonal relationship between therapist and client, including empathic understanding, level of regard, social bonding, and supportiveness. In the current analysis, we focus on social bonds and personal dimensions of the therapeutic alliance.

During the course of inpatient or outpatient treatment, patients often rely on mental health providers for medication management, psychotherapy, and discharge planning, or for access to other resources or services essential for recovery. However, estimates suggest that, of those with serious mental illness who have had contact with mental health services, nearly one-third withdraw from care (Kreyenbuhl *et al*. 2009). Given the high rates of patient drop-out and treatment non-compliance among this population, much research has been devoted to identifying characteristics of mental health consumers who disengage and the treatment providers that are able to promote positive health outcomes. Since the 1950s, a focus on the therapeutic alliance has yielded important insights, documenting the salubrious effects of a strong therapeutic alliance on markers of short- and long-term treatment outcomes (Rogers, 1951; Cruz & Pincus, 2002; McCabe & Priebe, 2004). For example, greater adherence to prescribed medication regimens (Curtis *et al*. 2010), improved continuity of care (Adair *et al*. 2005), symptom reduction (Adams *et al*. 2007), and better overall treatment and care satisfaction reports (Adams *et al*. 2007) are associated with continued and positive interactions between clients and providers. Not surprisingly, the converse has also been documented: the absence of a positive patient–provider relationship has been shown to result in perceptions of treatments as coercive and linked to treatment noncompliance or cessation (Johansson & Eklund, 2006; Roe *et al*. 2009).

On the one hand, research links a strong therapeutic alliance to provider and patient characteristics. Among providers, interpersonal sensitivity (Johansson & Eklund, 2006), warmth (as opposed to being perceived as cold or distant), involving patients in decision-making (Curtis *et al*. 2010; Drake *et al*. 2010), and respectful attitudes toward patients have been found to positively contribute to the therapeutic alliance (Ackerman & Hilsenroth, 2003; Hilsenroth *et al*. 2012). Among patients, negative attitudes toward treatment is associated with a weaker therapeutic alliance (Barrowclough *et al*. 2010), while a strong therapeutic alliance is built on feelings of patient trust in their practitioner. In addition, the nature of communication between these parties matters. Namely, more frequent and longer duration of contact with providers enhances the therapeutic alliance, driving positive outcomes (Sajatovic *et al*. 2004; Velligan *et al*. 2010; Hilsenroth *et al*. 2012).

On the other hand, though socio-demographic characteristics of providers or patients (e.g. gender, race/ethnicity) have been posited to influence the strength of the therapeutic alliance, the limited amount of research on gender, race, or other socio-demographic concordance has produced ambiguous results (e.g. Zlotnick *et al*. 1998; Givens *et al*. 2007; Cooper & Powe, 2004). Further, findings across studies indicate that the type of therapy provided has very little, if any, bearing on the therapeutic alliance (Salvio *et al*. 1992; Neale & Rosenheck, 1995; Horvath, 2001).

Taken together, research findings on the treatment of serious mental illness provide evidence that characteristics of patients, providers, and their dyadic relationship shape perceptions of the therapeutic alliance. In turn, the strength of the therapeutic alliance plays an important role in shaping mental health outcomes. Though this line of research represents an important contribution to the study of individual-level factors that improve recovery outcomes, it provides little insight into the processes *outside* the context of treatment that might influence the therapeutic alliance. Specifically, the therapeutic alliance is not formed in a vacuum. Much of this research lacks attention to the broader social environment in which patients are embedded outside the treatment context and in their everyday lives. *Specifically, we posit that perceptions of a client's relationships with health providers – their therapeutic alliance – are influenced by social interactions within informal networks in the community, including kin and friends.* If outside networks influence inside networks, the quality and characteristics of the larger social context in which clients are embedded may have important effects on the therapeutic alliance.

## Social networks and mental health services

The notion that community-based social networks matter for health has both a solid foundation and an ever-growing body of research exploring the dynamic interplay between community and treatment systems. However, the focus on the interaction of networks in the community and networks in the treatment system represents an understudied area (Pescosolido, 2006). The Network Episode Model (NEM) has emerged as a useful theoretical framework for understanding how networks operate and shape decision-making during the illness episode (Pescosolido, 1991; 1992; Pescosolido *et al*. 1998*a*, *b*). The key argument is that lay and professional social networks play a central role in influencing response to illness.

The NEM is a departure from past work that tends to conceptualize health related decision-making as the product of individual, rational actors operating under a strictly cost–benefit logic or as the byproduct of individuals’ socio-demographic location (i.e. education, socioeconomic status, gender) and illness severity. The NEM argues that an unfolding episode of illness is generally understood through an individual's interaction with community-based social ties that help them understand, recognize, and respond to the onset of changes in health (Pescosolido, 1992). Through interaction with family members, friends, and other confidants, individuals ascribe meaning to their symptoms, and determine what ameliorative actions are appropriate, when to pursue such action, and to what extent to accept and comply with prescribed treatments. In addition to recognizing behavioral change and the onset of symptoms, individuals living with mental illness rely on their informal ties for emotional support, instrumental support, and advice (Pescosolido, 1991; Pescosolido *et al*. 1998*a*, *b*).

Importantly, just as network ties shape attitudes and behaviors during an illness episode, they are, in turn, shaped by the experience of illness. Serious mental illness, especially its onset and management during periods of acute symptoms, represents a major disruption in routine daily life that can prompt a ‘network crisis’ (Lipton *et al*. 1981; Perry & Pescosolido, 2012). Research indicates that network members are selectively involved, whether that selection is done by the individual experiencing onset or by those around them, to manage health concerns, and their intervention may turn out to be helpful or harmful (Bolger *et al*. 2000; Rafaeli & Gleason, 2009; Perry & Pescosolido, 2015). Some recent research suggests a relationship between serious mental illness and social isolation from peers (Chou *et al*. 2011), but family members tend to remain involved and influential throughout the illness career (Biegel & Schulz, 1999; Fleury *et al*. 2008), and most clients prefer family support and integration (Cohen *et al*. 2013).

Initially social network researchers conceptualized networks as supporting formal treatment (Kadushin, 1966; Horwitz, 1977). But with conflicting findings, subsequent research focusing on the cultural context revealed that network characteristics matter, with some predisposing individuals to treatment while others impede it (Pachucki & Breiger, 2010; Perry & Pescosolido, 2015). Individuals may be consciously or unconsciously socialized within the context of their lay networks to distrust health care providers, like psychiatrists and other physicians, thereby discouraging formal treatment seeking or the establishment of a strong therapeutic alliance (Lo & Stacey, 2008). For example, Pescosolido *et al*. (1998*a*, *b*) found that larger, more supportive networks tended to decrease the use of formal health care providers in Puerto Rico where cultural norms see the need for psychiatric treatment as a failure of the family (see also Faccincani *et al*. 1990; Aberg-Wistedt *et al*. 1995; Becker *et al*. 1997). Moreover, highly supportive community ties can substitute for the typical functions of treatment providers, providing advice and information, emotional support, and regulating medication compliance in ways that diminish perceived need for formal services (Carpentier & Bernard, 2011; Thoits, 2011). This could lead to a pattern wherein stronger lay support networks are associated with less positive evaluations of resources provided by clinicians.

Research also suggests that the way advice or support is provided may contribute to individuals’ perceptions of formal mental health care. Informal ties that coerce individuals with serious mental illness into treatment whether they are interested in formal services or not can contribute to negative feelings or skepticism toward providers (Rogers, 1993; Pescosolido *et al*. 1998*a*, *b*). This can have long-term effects on mental health outcomes, threatening the establishment of a strong therapeutic alliance, discouraging consistent compliance with prescribed treatment, and leading to termination of mental health services.

Finally, consistent with a community support systems approach (Stroul, 1989; Tarrier *et al*. 1989; Pitschel-Walz *et al*. 2001), a strong social safety net in the community may provide an environment that is conducive to the development of attachment to one's therapist and more positive feelings about treatment more broadly. Evidence-based interventions such as family psychoeducation, for example, are designed to facilitate collaborative management of people with SMI by professionals and family members, including coordinating treatment, ensuring medication compliance, and involving family members in treatment planning (Lucksted *et al*. 2012). These programs also emphasize the importance of minimizing family conflict and improving communication and problem solving. In other words, key objectives of programs that involve the family are to: (1) cultivate broader community support for treatment goals; and (2) create a healthy social environment in the community that is conducive to recovery. It stands to reason that even in the absence of an intervention like family psychoeducation, strong and amicable relationships with lay community members may reflect broader support for treatment decisions and positive social functioning, which translate into more positive perceptions of providers among individuals with SMI and their lay community networks.

## The present study

Overall then, existing research indicates that it is not simply the presence or absence of community ties alone that shape the influence of informal social networks on health services experiences and actions. Rather, community network closeness, composition, or culture may play a significant role in informing the attitudes of individuals with serious mental illness toward mental health services and their treatment providers. Thus, whether community social networks and treatment social networks are in sync or clash in prescriptions and proscriptions represents an essential part of the dynamic that shapes personal dimensions of the therapeutic alliance (Pescosolido & Boyer, 2010).

Further, the efficacy of formal health care providers in the recovery process may depend on the types and amount of resources that are embedded in the community-based network. Although no research to date has explicitly examined how characteristics of outside (i.e. community) social networks shape the inside networks, including interpersonal aspects of the therapeutic alliance like perceptions of supportiveness, these questions are critically important. Identifying the full set of factors contributing to meaningful engagement with formal mental health care providers is essential for understanding persistence in treatment and the role of formal and informal caregivers in recovery.

The present study aims to achieve a better understanding of the interactions between provider and social network characteristics in influencing perceptions of the interpersonal therapeutic alliance. We ask three research questions grounded in key supportive and affective elements of the therapeutic alliance: listening when the client is upset, providing helpful advice and information, and expressing care and concern. These research questions are: (1) How do evaluations of treatment providers, including perceptions of listening, advising, and caring, compare with those of other members of the social network?; (2) Focusing on dyadic relationships, how do individual and relationship characteristics influence perceptions of providers and lay community members as people who listen, advise, and care?; and (3) Looking to the network context, how are properties of social networks as a whole associated with perceptions of providers and community members as people who listen, advise, and care?

## Data and methods

### Sample

The INMHS, fielded between 1990 and 1997, remains as one of the few mental health studies of ‘first timers’ using a detailed egocentric network methodology to identify relationships between the early mental illness career and social network dynamics (additional information at http://www.indiana.edu/~icmhsr/inmhs.html). The INMHS was conducted at two large public and private hospitals and a hospital-affiliated community mental health center. New patients to the facilities were asked to participate in face-to-face interviews if they met the following inclusion criteria: (1) 18 years or older, (2) first major contact with the mental health treatment system, and (3) mental illness history of no longer than 2 years. These inclusion criteria were assessed using self-report. Participants were administered the Structured Clinical Interview for DSM-III-R (Spitzer *et al*. 1990) and recruited if a major Axis I research diagnosis was identified (schizophrenia, bipolar disorder, or major depression). A comparison sample with less serious mental illness (largely adjustment disorder) was also recruited simultaneously. A total of 66.4% of individuals asked to be in the INMHS consented.

The sample is composed of 173 individuals (i.e. egos or individuals making contact) who participated in the first round of face-to-face interviews, conducted within 3 months of treatment initiation. These egos provided information about 2575 members of their community-based network (i.e. alters), including friends, family members, coworkers, neighbors, mental health treatment providers, and others. However, four ego respondents and 322 alter observations were dropped due to missing data on key study variables, leaving an effective sample size of 169 egos and 2253 alters. Because of the complexity of levels and characteristics, the more specialized terminology of ego, alter and whole network is necessary from this point for clarity, even as these terms are generally unfamiliar outside of network science.

Table 1 reports characteristics of the sample. Overall, 65% of respondents in the full sample are female; 73% are white and 27% are black. Respondents’ average education is 11.57 years. About 52% of respondents are diagnosed with major depression, 7% with bipolar disorder, 13% with schizophrenia, schizoaffective disorder or similar, and 28% with less severe disorders (largely adjustment disorder).

**Table 1.** Sample descriptive statistics, Indiana Network Mental Health Study

	*N*	Proportion	Mean	s.d.
Ego characteristics (*n* = 169)				
Female ego	109	0.65		
White	124	0.73		
Education (years)			11.57	2.00
Diagnosis				
Bipolar	12	0.07		
Schizophrenia	22	0.13		
Major depression	88	0.52		
Other	47	0.28		
Alter characteristics (obs = 2206)				
Female alter	1252	0.57		
Relationship type				
Partner/spouse	87	0.04		
Parent	217	0.10		
Sibling	325	0.15		
Other kin	594	0.27		
Friend	475	0.22		
Mental health provider	209	0.09		
Other	299	0.13		
Very close	955	0.43		
Frequent contact	1108	0.50		
Ever hassles	578	0.26		
Support functions				
Listens	1536	0.70		
Advises	1173	0.53		
Cares	1347	0.61		
Whole social network characteristics (*n* = 169)				
Percent female			55.20	14.38
Percent kin			56.23	17.35
Percent friends			22.09	15.16
Percent MH providers			11.47	9.77
Mean closeness			2.20	0.35
Mean frequency of contact			2.33	0.31
Mean hassles			1.40	0.30
Network size			21.19	10.10

### Measures

#### Name generators

As noted above, the INMHS-collected data on respondents’ social network ties or alters across a broad range of social domains (e.g. work/school, household, clinic, and church). Each domain has a corresponding name generator (i.e. a question which asks patients to list their network ties in that social context or with particular characteristics), with no limitations on the number or types of people respondents could name. These include: contacts during the illness episode, household members, partner/spouse/boy or girlfriend, family members in regular contact, coworkers, classmates, fellow volunteers, close friends, casual friends, enemies, important matters discussants, health matters discussants (Perry & Pescosolido, 2010), people with similar problems, significant hospital/clinic staff and treatment providers, and people who help or hinder medication compliance. This approach provides a near-complete inventory of people who have semi-regular contact with respondents, including ties of great significance as well as those who may play a more peripheral or unidimensional role (e.g. neighbors and coworkers).

#### Measures

To capture the complexity of individuals nested in dyadic relationships and social networks, variables are measured at three levels. First, the individual level of clients is addressed. Socio-demographic characteristics like gender, race, age, and educational attainment have been the focus of therapeutic alliance research and may shape the structure and function of personal community networks (Marsden, 1987; Ajrouch *et al*. 2001; Peek & O'Neill, 2001). These variables are considered as controls in regression analyses. Gender (1 = female; 0 = male) and race (1 = white; 0 = black) are coded into dummy variables. Mental illness diagnosis is coded into four dichotomous indicators representing: (1) bipolar disorder, (2) major depression, (3) schizophrenia, schizoaffective disorder, or Psychosis NOS, and (4) a group of less severe other disorders comprised largely of respondents with adjustment disorder. Socioeconomic status is measured using years of education.

Other controls were included in initial models (e.g. age and income), but these were removed from final models due to non-significance and in the interest of parsimony. We also consider the potential confounding effects of self-esteem [Rosenberg (1965) self-esteem scale], sociability (two binary variables measuring whether respondents typically talk to others about their problems and mind when others talk to them about their own problems, respectively), and satisfaction with social relationships (a summed scale comprised of 12 items asking how the respondent feels on a delightful-terrible response metric about social relationships across a variety of domains, including family, household members, friends, coworkers, and treatment providers). Results from these models are not presented in tables because they do not differ substantively from the restricted models.

Second, at the relationship level, we measure the type of tie between the client and each member of the network. Specifically, a series of dichotomous variables representing partner/spouse, parent, sibling, other relative, friend, mental health treatment provider, and other type of relationship are considered. For some analyses, these are collapsed into four categories – kin, friends, mental health providers, and others. In addition, relationship closeness, frequency of verbal or face-to-face contact, and hassles or causes problems are dichotomized to ‘very’ close, contact that occurs ‘often,’ and ever hassling or causing problems (the original categories of ‘sometimes’ and ‘often’ are combined because of small cell sizes). Interpersonal and supportive functions are included in models as binary dependent variables and include whether the alter: (1) ‘Tells you they care about what happens to you;’ (2) ‘Gives you suggestions when you have a problem about what you could do, where you could go, or who you could talk to;’ and/or (3) ‘Listens to you when you are upset or down.’ These are coded 1 for ‘yes’ and 0 for ‘no.’ Finally, alter gender is included at this level as a control, and is binary (1 = female; 0 = male).

Third, at the social network level, measures of whole network characteristics include percent women, percent kin, percent friends, and percent mental health treatment providers (expressed in units of 10%). Average closeness, frequency of contact, and hassles are simply the mean of reported closeness, contact, and trust on the three-point Likert scales described above, reported for each alter and aggregated across the total network. Finally, all models control for network size (total number of alters named by each ego).

### Analysis

To address the first research question, we begin with a simple description of the role and evaluation of providers in the lives of individuals who are in treatment for a mental health problem using bivariate statistics. For the second research question, we identify characteristics of clients, alters, and relationships that are associated with perceived social resources at the dyadic level (i.e. whether each alter is evaluated as someone who listens, advises, and/or expresses care and concern). Additionally, we use interaction models to determine whether individual and relationship characteristics are more or less predictive of perceptions of the personal therapeutic alliance with providers relative to evaluations of lay supporters. To examine the third research question, we determine whether aggregate characteristics of social networks are associated with perceptions of listening, advising, and caring. We then use interaction models to determine whether perceptions of interpersonal dimensions of the therapeutic alliance with providers are associated with network characteristics to a greater or lesser degree than evaluations of interpersonal resources offered by lay supporters. Finally, to examine potential confounding social or personality traits that might explain the relationship between network characteristics and perceptions of the therapeutic alliance, we conduct a series of *post hoc* sensitivity analyses controlling for measures of self-esteem, sociability, and satisfaction with social relationships.

Multilevel regression modeling uses data on mental health clients (egos) and alters at the point of entry into mental health treatment (Wave 1 of INMHS only). Specifically, a two-level random-intercept model is used with level-1 alters (the client's set of ties) nested in level-2 egos (the clients). These models include a random intercept for each ego, and adjust for the lack of independence between alter observations nested within egos. The two-level binary logistic regression model predicting probability *p* of ego *j* reporting that alter *i* provides a support resource is written as:
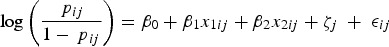


In this model, *i* corresponds to alter identifier (level 1), *j* to ego identifier (level 2), *ζ*_*j*_ to the random intercept at the ego respondent level, and *ε*_*ij*_ to the level-1 residual. Together, *ζ*_*j*_ and *ε*_*ij*_ represent random parts of the model, while the other components are fixed.

This analytic strategy is ideal in cases where the dependent variable is a characteristic of alters or ties since aggregation to the ego level results in a loss of information. In all multivariate models, we prevent cluster confounding (Seaman *et al*. 2014) by including contextual variables (i.e. aggregated versions of alter characteristics) for all level-1 measures that vary within egos (e.g. alter gender and percent of alters who are female are both included). Interactions are detected using pooled regression models with an interaction term. Significance of interactions is determined using Chow-type tests of the equality of coefficients and is confirmed using the Delta method for differences in predicted probabilities. All significant interactions are presented as figures of predicted probabilities rather than in tables to facilitate interpretation. All regressions control for ego characteristics and level-1 and -2 versions of constitutive terms in the interaction model.

## Results

As shown in Table 1, the size of the total network of regular interaction partners is about 21.19 alters, on average. About 70% of alters are reported to listen to respondents when they are upset, 53% provide advice and information, and 61% tell them that they care what happens to them. In total, 77% of mental health clients named a treatment provider as someone they interact with semi-regularly. However, only 9% of mental health clients’ alters are treatment providers, suggesting that most people name only one (32%) or two (20%) providers to their network. Other characteristics of clients and their social networks are presented in Table 1.

### Question 1: Comparison of treatment providers and lay community network ties

Table 2 provides a comparison of perceived characteristics of treatment providers (i.e. ‘inside’ networks) and members of the lay community (i.e. outside’ networks). Treatment providers are more likely to be women than lay network members (65% *v*. 55%, *p* < 0.01). Also, clients report feeling very close to nearly half (46%) of lay supporters, but to only 26% of treatment providers (*p* < 0.001). They also indicate more frequent contact with lay network members relative to treatment providers (52% *v*. 36%, *p* < 0.001). However, with respect to support functions, providers are evaluated as well or better than lay members of the network. For example, 81% of treatment providers and 68% of lay supporters listen to the client when they are upset (*p* < 0.001). Likewise, 66% of providers and about half of lay network members (52%) are reported to provide useful advice and information (*p* < 0.001). Finally, although clients are slightly less likely to report that providers care about what happens to them in comparison with lay people (57% *v*. 62%), this difference is not statistically significant.

**Table 2.** Comparison of treatment providers and lay members of community social networks, Indiana Network Mental Health Study (N = 2206)

	Treatment providers		Lay network members		
	*N*	*Proportion*	*N*	*Proportion*	*χ*^2^
Female	191	0.65	1217	0.55	9.65**
Very close	72	0.26	983	0.46	40.23***
Frequent contact	98	0.36	1116	0.52	25.94***
Ever hassles	45	0.23	570	0.29	2.72
Support functions					
Listens	169	0.81	1369	0.68	13.71***
Advises	138	0.66	1035	0.52	15.32***
Cares	120	0.57	1229	0.62	1.32

*, *p* < 0.05; **, *p* < 0.01; ***, *p* < 0.001.

**Fig. 1.**
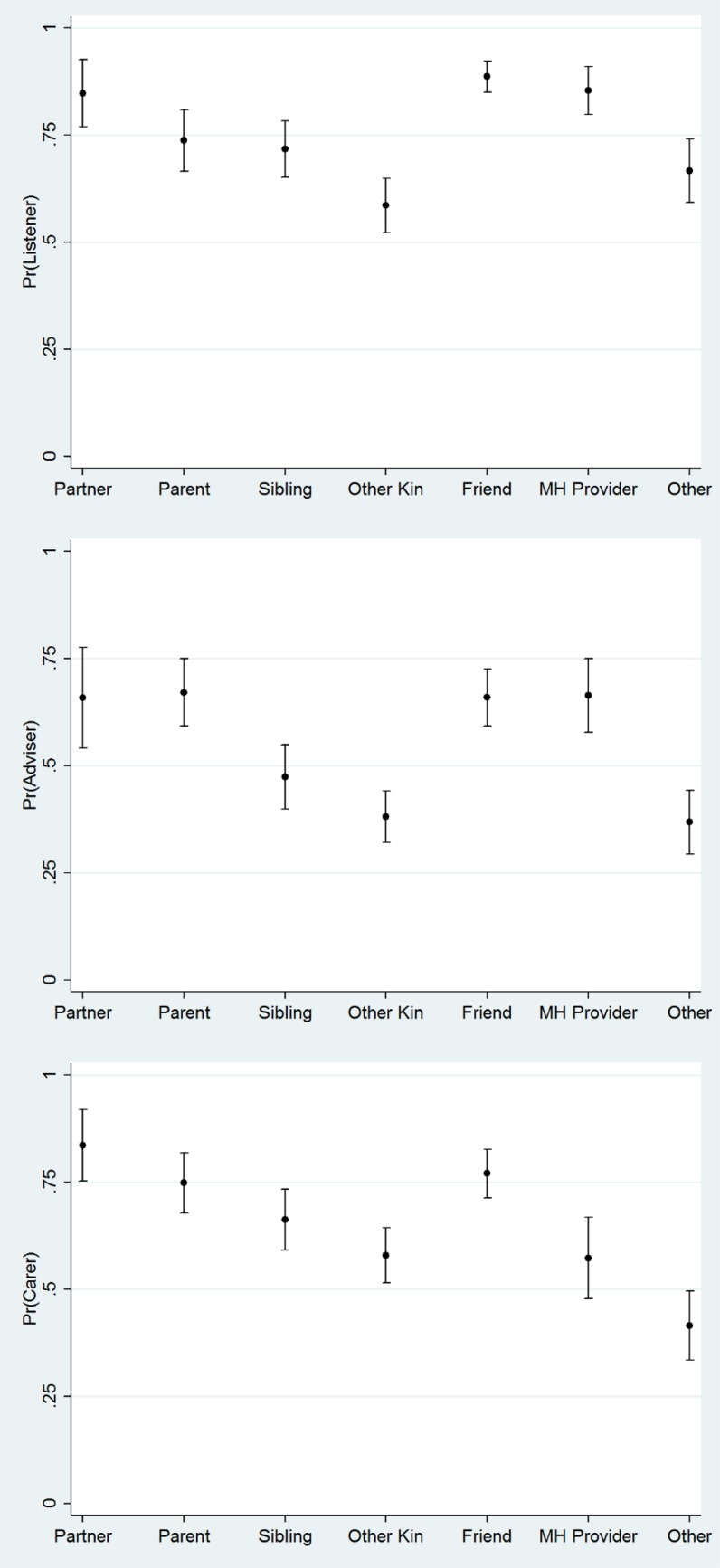
Predicted probability of support functions by relationship to ego, Indiana Network Mental Health Study.

Figure 1 displays predicted probabilities to highlight mental health clients’ perceptions of support functions provided by their mental health treatment providers relative to different types of lay members of the network. These are based on regression models that control for all ego characteristics, as well as the percent of the whole social network comprised of kin, friends, providers, and other types of ties. The likelihood of reporting that treatment providers listen when the respondent is upset and that they provide advice and information is on par with levels reported for spouses/partners, parents, and friends. Moreover, treatment providers are significantly more likely than siblings, other kin, and other types of ties to be perceived as listeners and advisers. With respect to expressing care and concern for the client, partners, parents, and friends are significantly more likely to fulfill this function than treatment providers. However, perceptions of caring among providers are similar to and not significantly different from siblings and other kin. Overall, perceptions of the role of treatment providers in offering crucial support are positive among this sample of clients.

### Question 2: Effects of individual and relationship characteristics on perceptions of listening, advising, and caring

As shown in Table 3, characteristics of alters are associated with perceptions of the support functions of social network members. Regression coefficients confirm the findings on the type of relationship, also presented in predicted probabilities above (see Fig. 1). Namely, mental health treatment providers are more likely than other types of non-kin ties to listen when the person with mental illness is upset (OR = 2.768; *p* < 0.001) and provide advice and information (OR = 2.837; *p* < 0.001), on average, but are no more likely to tell the person that they care about them. In addition, alters who are women – both treatment providers and lay people – are disproportionately like to be perceived as good listeners (OR = 1.648; *p* < 0.001), providers of informational support (OR = 2.837; *p* < 0.001), and as caring (OR = 1.528; *p* < 0.001) relative to men.

**Table 3.** Random-intercept logistic regression results for the effects of ego, alter, relationship, and network characteristics on perceived support functions, Indiana Network Mental Health Study (obs = 2206, n = 169)

	1. Listens		2. Advises		3. Cares	
	OR	CI	OR	CI	OR	CI
Ego characteristics						
Female	1.861**	(1.101–3.145)	0.918	(0.548–1.540)	1.864**	(1.084–3.207)
White	1.319	(0.770–2.260)	0.934	(0.552–1.582)	1.392	(0.795–2.438)
Education (years)	1.022	(0.910–1.148)	1.040	(0.927–1.166)	1.113*	(0.986–1.256)
Diagnosis						
Bipolar	1.094	(0.430–2.779)	1.496	(0.607–3.686)	1.580	(0.598–4.172)
Schizophrenia	1.011	(0.468–2.184)	1.825	(0.854–3.904)	1.170	(0.526–2.601)
Major depression	0.787	(0.470–1.320)	1.344	(0.804–2.244)	1.003	(0.586–1.717)
Alter/tie characteristics						
Relationship to ego						
MH provider	2.768***	(1.672–4.584)	2.837***	(1.865–4.315)	1.317	(0.860–2.017)
Kin	0.706**	(0.502–0.992)	1.206	(0.887–1.640)	2.007***	(1.441–2.797)
Friend	2.203***	(1.449–3.348)	2.272***	(1.599–3.229)	2.779***	(1.901–4.065)
Female	1.648***	(1.297–2.094)	1.337***	(1.082–1.652)	1.528***	(1.216–1.922)
Very close	4.657***	(3.442–6.302)	2.445***	(1.904–3.140)	4.718***	(3.570–6.234)
Frequent contact	1.521***	(1.161–1.993)	1.303**	(1.029–1.650)	1.581***	(1.225–2.041)
Ever hassles	0.575***	(0.433–0.762)	0.669***	(0.518–0.864)	0.768*	(0.585–1.010)
Social network characteristics						
Percent MH providers	0.924	(0.727–1.174)	0.908	(0.716–1.151)	0.865	(0.673–1.111)
Percent kin	0.936	(0.786–1.115)	1.011	(0.851–1.203)	0.927	(0.773–1.112)
Percent friends	1.102	(0.918–1.322)	1.042	(0.871–1.245)	0.972	(0.806–1.172)
Percent female	0.846*	(0.716–1.001)	0.894	(0.757–1.054)	0.815**	(0.684–0.972)
Mean closeness	3.739***	(1.699–8.227)	1.149	(0.530–2.488)	3.078***	(1.356–6.986)
Mean frequency of contact	0.454*	(0.195–1.057)	1.487	(0.647–3.417)	0.718	(0.300–1.719)
Mean hassles	1.369	(0.613–3.057)	1.370	(0.622–3.015)	0.705	(0.307–1.620)
Network size	1.001	(0.978–1.024)	1.015	(0.992–1.038)	0.997	(0.973–1–021)
ICC	0.26		0.28		0.30	
Wald *χ*^2^	249.69***		153.42***		256.28***	

*, *p* < 0.05; **, *p* < 0.01; ***, *p* < 0.001.

Table 3 also indicates that properties of dyadic relationships are correlated with perceptions of alters. Relationships characterized by closeness are significantly more likely to be associated with perceptions of listening (OR = 4.657; *p* < 0.001), providing advice (OR = 2.445; *p* < 0.001), and caring (OR = 4.718; *p* < 0.001). Consistent with previous research, more frequent contact is also associated with perceptions of listening (OR = 1.521; *p* < 0.001), advising (OR = 1.303; *p* < 0.001), and caring (OR = 1.581; *p* < 0.001). In contrast, alters that are reported as sometimes or often hassling, causing problems, or making life difficult are significantly less likely to be labeled as listeners (OR = 0.575; *p* < 0.001), advisers (OR = 0.575; *p* < 0.001), or people who care (OR = 0.768; *p* < 0.05).

To this point, absent significant interactions between being a treatment provider and alter and relationship characteristics, we can conclude that being a woman, having a closer bond, and being in more frequent contact are associated with more positive perceptions of support provided by clinicians and lay people. However, there are significant (*p* < 0.05) interactions between being a provider and ‘hassling’ such that the effects of causing problems or making life difficult are stronger among mental health professionals than lay people on perceived listening (OR = 0.110 *v*. OR = 0.482), advising (OR = 0.258 *v*. OR = 0.598), and caring (OR = 0.263 *v*. OR = 0.678). In other words, *the effect of conflict and negativity is disproportionately detrimental for mental health treatment providers, and is significantly associated with perceptions of support.*

### Question 3: Effects of social network characteristics on perceptions of listening, advising, and caring

Whole social network properties are also found to be related to perceived support functions of alters, controlling for the tie characteristics, as seen in Table 3. The larger the proportion of women in a network, the less likely any given alter embedded in that network is to be perceived as a listener (OR = 0.846; *p* < 0.05), net of that alter's own gender. The same relationship between gender composition of the social network and perceived caring is also observed (OR = 0.815, *p* < 0.01). Alters embedded in networks with higher mean closeness have significantly greater odds of being perceived as good listeners (OR = 3.739; *p* < 0.001) and as caring (OR = 3.078; *p* < 0.001). In contrast, alters embedded in networks with a greater mean frequency of contact are significantly less likely to be perceived as listeners (OR = 0.454; *p* < 0.05).

**Fig. 2.**
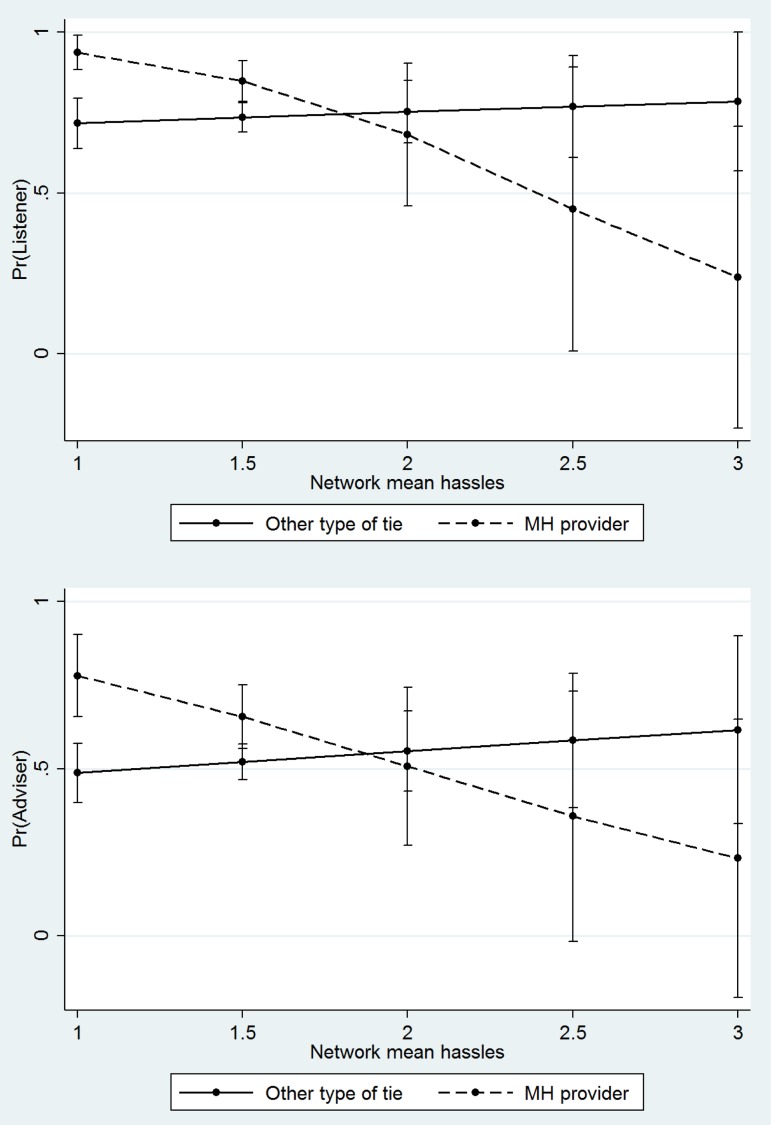
Predicted probability of support functions by mean network hassles and provider status, Indiana Network Mental Health Study.

Figure 2 reveals the relationships between the broader network context and perceptions of treatment providers relative to lay members of the network. Results are based on models that include interactions between being a provider and network characteristics. Importantly, as the network as a whole becomes more contentious and characterized by conflict (as measured by average hassling, causing problems, and making life difficult across network members), perceptions of treatment providers as good listeners and advisers are increasingly negative. However, when there is little conflict in the social network, treatment providers are perceived as being especially likely to provide support functions. In contrast, the level of conflict and hassling in the network is not significantly associated with perceptions of the support provided by other types of network ties. It is important to note that these interaction models control for each alter's own level of hassling. In other words, *the level of conflict in community-based networks is negatively associated with perceptions of treatment providers even if they are not themselves labeled as someone who hassles or makes life difficult.*

**Fig. 3.**
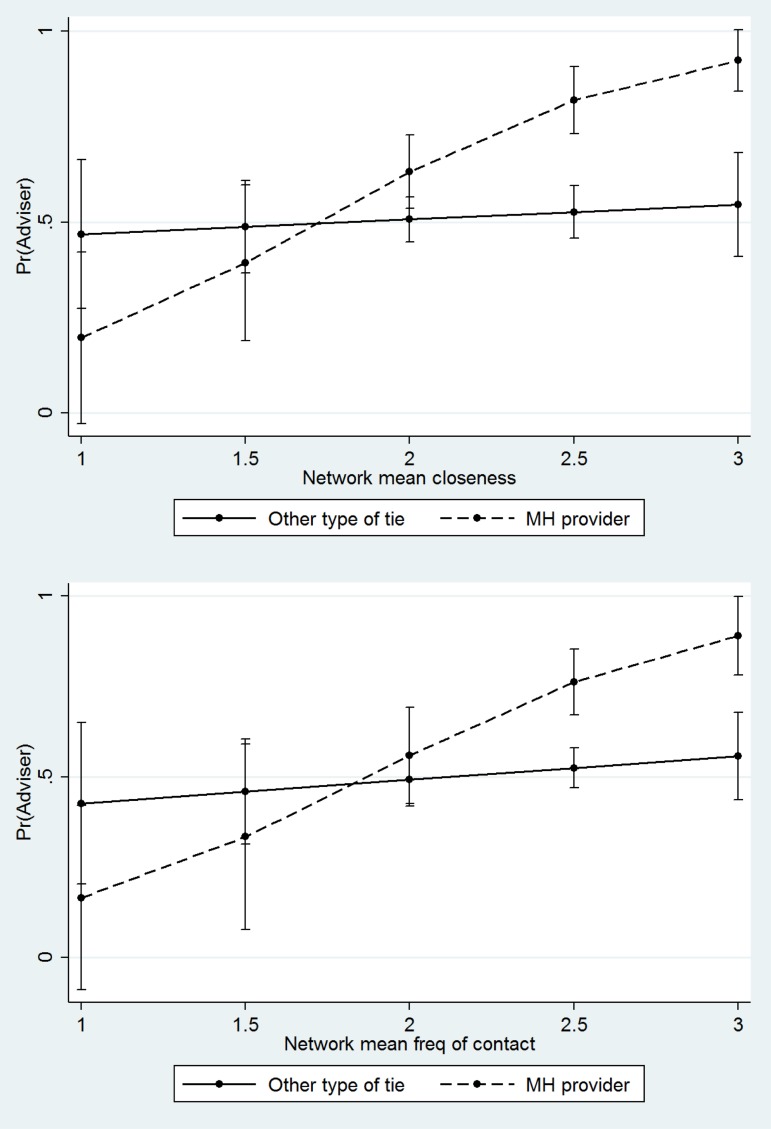
Predicted probability of support functions by mean network closeness, frequency of contact, and provider status, Indiana Network Mental Health Study.

As shown in Fig. 3, perceptions of mental health treatment providers as advisers are also correlated with the strength of ties in the network as a whole. Among clients embedded in community networks characterized by closeness and regular contact, perceptions of treatment providers are more positive. In contrast, the strength of the whole social network has no significant relationship to perceptions of lay people as advisers. These interaction models also control for closeness and frequency of contact with the provider him or herself. Thus, *net of the dyadic relationship with the provider, when the informal safety net is strong, individuals are especially likely to report that their treatment providers offer useful advice and information.*

Finally, to assess the potential confounding effects of social and personality traits such as self-esteem, sociability, and satisfaction with relationships, sensitivity analyses were conducted adding these variables as controls to the interaction models above. In other words, it is possible that some trait-like attachment (i.e. willingness and ability to establish and maintain positive relationships), extroversion, extent of social withdrawal, or similar might influence both reports about relationships with lay members of the network and perceptions of the therapeutic alliance. We added self-esteem, variables measuring sociability (tendency to talk to others and feelings about being talked to), and satisfaction with social relationships across 12 domains to interaction models. In general, sociability variables and relationship satisfaction were significantly related to perceptions of listening, caring, and advising (results available upon request). However, these variables did not diminish the strength or significance of the relationship between network context and perceptions of resources offered by treatment providers.

## Discussion

Our concern for understanding the role of social relationships in an individual's response to the onset of mental illness integrates two traditional questions in mental health services research: (1) What is the influence of social networks in the community on how individuals recognize mental health problems and respond with help-seeking?; and (2) How do social relationships in the treatment system shape process and outcomes for individuals in need? We reconceptualized these questions in terms of their intersection. That is, is the larger social context in which individuals live associated with perceptions of treatment providers, including interpersonal elements of the therapeutic alliance? Translated into network science terms: How are social networks ‘outside’ of the treatment context related to social networks ‘inside’ it (Pescosolido, 2006)?

Specifically, we asked three questions about the interface of community and treatment systems relevant to the therapeutic alliance. First, how likely are mental health clients to endorse positive evaluations of key elements of the personal therapeutic alliance – listening when they are upset, providing useful advice and information, and expressing care and concern – compared with other lay members of the social network? Second, focusing on dyadic relationships, how are individual and relationship characteristics associated with perceptions of providers and lay community members as people who listen, advise, and care? Third, looking to the network context, how are properties of social networks as a whole related to perceptions of providers and community members as people who listen, advise, and care?

The answers are fairly straightforward. Most individuals list at least one provider as part of their social network, though providers only make up a small proportion of clients’ networks. Moreover, evaluations of providers are favorable, even in comparison to personal ties in the community; and individual and relationship characteristics associated with perceptions of listening, advising, and caring (e.g. closeness, frequency of contact) are in line with previous research on the therapeutic alliance. However, as predicted by the NEM, the intersection of social networks and relationships inside and outside the treatment system is critical. Specifically, where clients’ community-based social network is strong, individuals are especially likely to report that their treatment providers offer useful advice and information. However, the level of conflict in the broader social network is negatively related to favorable perceptions of treatment providers, even if they are not themselves labeled as someone who hassles or makes life difficult. Further, the effect of conflict and negativity in the outside network is disproportionately detrimental for mental health treatment providers, and is significantly associated with poorer perceptions of the interpersonal therapeutic alliance and clinician supportiveness.

In sum, the lives of individuals inside and outside of the treatment system are, as we might expect, intimately intertwined. However, in research, treatment, and policy, we have tended to think of the community and the treatment site as distinct, encapsulated institutions. Prior research has examined how community systems affect entry into the treatment system. Our findings go further, suggesting that community systems may have a fundamental influence on how individuals experience the treatment system and the therapeutic alliance, in particular. Our findings are consistent with the influence of networks ‘outside’ the treatment system reverberating on networks ‘inside’ the treatment system. By extension, outside networks may shape an individual's return to the community and their likelihood of recovery both directly through support or conflict, and indirectly through perceptions of supportive elements of the therapeutic alliance. However, we wish to emphasize the speculative nature of this interpretation given our inability to definitively establish causation.

This study does have limitations, including the relatively small sample size and the collection of data prior to the passage of the Patient Protection and Affordable Care Act. Likewise, these data were collected prior to the emergence of social media and online support groups as a source of network support and social interaction for people with SMI (Álvarez-Jiménez *et al*. 2012). It is unknown how online community networks might influence client perceptions of the therapeutic alliance. In addition, because we did not use a prospective longitudinal design, it is not possible to determine causation, though we did attempt to address potential confounding effects of social and personality traits using controls. Finally, data on task-related dimensions of the therapeutic alliance are unavailable, making it impossible to determine whether these findings extend to other important elements of this relationship, including goal agreement. Future research should address these limitations using newer data.

Despite its limitations, the INMHS of first-time mental health clients is unique in its detailed assessment of characteristics of network ties across both community and treatment contexts. Specifically, the INHMS asks about relationships of distinct types (e.g. at work, in the household, in the clinic/hospital) and of different valences (e.g. supporters, advisors, casual acquaintances, and hasslers). Moreover, the INMHS assesses client evaluations of the functions provided by each network tie (e.g. good at listening, caring), including the providers they list. This allows a conceptualization and analysis that matches the complexity of the real life situation that clients, their providers, and their social ties face. Likewise, it crosses disciplinary boundaries that have traditionally conceptualized and measured the therapeutic alliance as independent from the broader network environment. Given the findings presented here, it may be time to revisit how we understand what happens to individuals as they experience the onset of mental illness and to reconsider the research designs and models of care that we employ.

To date, community support systems approaches, consumer-centered family models, and related evidenced-based interventions for families (e.g. psychoeducational therapy) have made important contributions to the development of more holistic and inclusive treatment models that embrace the role of lay supporters and community integration (Lucksted *et al*. 2012). However, we feel strongly that results presented here require a rethinking of the role of lay community networks in recovery in two significant ways. First, our findings underscore the need to conceptualize community networks more broadly to include relationships outside the family unit. Fewer than 30% of the network members with whom respondents regularly interacted were close family members (i.e. partners, parents, siblings, and children). An additional 27% were extended kin who are unlikely to be included in family-based interventions, and nearly half were friends and other non-kin. Yet, characteristics of these networks shaped perceptions of treatment providers in critical ways illustrating the influence of a larger and less tightly connected community safety net (i.e. what network scientists would call weak ties).

Second, our findings have implications for the scope of influence held by lay members of clients’ social networks. Existing community and family-based models of care tend to focus on the following core elements of recovery: (1) the effects of lay relationship quality and communication on clients’ wellbeing and clinical outcomes; (2) reduction of caregiver distress to improve support provision and help cope with challenges to the family system; (3) knowledge and planning of treatment goals, services coordination, and crisis intervention on behalf of the person with mental illness (Dixon *et al*. 2001; Pitschel-Walz *et al*. 2001). By and large, the emphasis remains on developing a social safety net that is conducive to recovery and supportive of the client's treatment goals. However, the extent to which conflict and support in community networks might undermine or strengthen the therapeutic alliance is seldom addressed. In other words, the ‘reach’ of community and family systems may infiltrate the therapeutic dyad. Additional research is needed to identify the specific mechanisms, but it is possible that recovery optimism and support conveyed by strong community networks subconsciously shape clients’ perceptions of resources provided by clinicians.

This research was conducted in the USA, but findings from international mental health services research are suggestive of similar patterns in other countries. For example, Canada, Israel, Australia, China, and all countries in Western and Central Europe have experienced deinstitutionalization and community mental health system reform that shares many similarities with the American experience (Becker & Vázquez-Barquero, 2001; Chien & Norman, 2003; Sealy & Whitehead, 2004; Rosen 2006; Abramowitz *et al*. 2008). Availability of acute and longer-term inpatient beds has decreased markedly in the past three decades as community mental health treatment systems have been expanded. However, as reform efforts have lost momentum, governmental support and funding for community mental health systems have declined and become inadequate to support people with serious mental illness in the community. At the same time, as the goals of mental health policy have shifted toward community reintegration, informal sources of support have become more critical for people with serious mental illness (Fakhoury & Priebe, 2002; Priebe *et al*. 2008). Despite increasing caregiving burdens on families and communities, benefits and support resources provided by health and social services to lay caregivers in countries that have experienced deinstitutionalization are almost universally insufficient, and there is little attempt to integrate professional and lay systems of care. Consequently, as in the USA, this creates clinical conditions wherein community networks are essential to the success of mental health services, but treatment providers have inadequate communication with family and other lay network members and little influence over clients’ social environments. This fragmented system of care is exactly the type of environment in which characteristics of lay social networks are likely to shape clients’ perceptions of the therapeutic alliance and support resources provided by clinicians.

In this era of translational science and personalized medicine, findings such as we document here reveal the community and the treatment sites as complex, interacting systems. This aligns with complexity theory (Ostrom, 2009), and requires that we reconfigure our understandings of how inside and outside social networks shaping the lives of person with mental illness are either mutually reinforcing or mutually destructive. This perspective – focusing on social networks and their interactions – pushes the boundaries of contemporary conceptualizations of community and community-based care for individuals with mental illness. The recovery potential for individuals cannot be divorced from the realities of the social connections on which they can rely both in and away from formal care.
